# Correlation of dermoscopic and histopathological features in basal cell carcinoma using computerized image analysis

**DOI:** 10.3389/fmed.2025.1581601

**Published:** 2025-04-30

**Authors:** Gökhan Kaya, Kübra Ataman, Sevgi Güleşçi, Ayşegül Yabaci Tak

**Affiliations:** ^1^Department of Dermatology, Ministry of Health Nizip State Hospital, Gaziantep, Türkiye; ^2^Department of Pathology, Ministry of Health, Kirikkale High Specialization State Hospital, Kırıkkale, Türkiye; ^3^Independent Artificial Intelligence Engineer, Istanbul, Türkiye; ^4^Department of Biostatistics and Medical Informatics, Bezmialem Vakif University, Istanbul, Türkiye

**Keywords:** basal cell carcinoma, dermoscopy, histopathology, artificial intelligence, skin cancer diagnosis, tumor depth, pigmentation patterns, dermatological imaging

## Abstract

**Background:**

Basal cell carcinoma (BCC) is the most common skin cancer, exhibiting local invasiveness despite its low metastatic potential. Dermoscopy and histopathology are essential for diagnosis, while quantitative assessments may enhance lesion characterization.

**Aim of the Study:**

This study aims to analyze the dermoscopic and histopathological characteristics of BCC and investigate the correlation between dermoscopic pigmentation patterns and tumor depth to improve lesion classification and diagnostic accuracy.

**Patients and methods:**

This retrospective study analyzed 41 patients with 42 histopathologically confirmed BCC lesions, evaluated at Nizip State Hospital and 25 Aralik State Hospital between April 2023 and February 2025. High-resolution dermoscopic images were analyzed alongside histopathological findings. AI-assisted computerized image analysis was employed to quantify lesion size and pigmentation percentage, while tumor depth and dermoscopic-histopathological correlations were manually assessed.

**Results:**

BCC was more prevalent in males (56.1%) and older adults, with a mean age of 67.1 years. The most commonly affected site was the nose (42.9%), followed by the cheek (14.3%) and upper lip (11.9%). Histopathologically, nodular (28.6%) and adenoid (28.6%) BCC were the most frequent subtypes. Dermoscopic analysis revealed blue-gray ovoid nests (57.14%) and arborizing telangiectasias (71.43%) as predominant features, particularly in mixed-type BCC, while blue-gray dots and globules (57.14%) were most common in micronodular BCC. Ulceration (45.24%) and multiple erosions (57.14%) were strongly associated with infiltrative BCC. A negative correlation was observed between pigmentation percentage and tumor depth, with deeper tumors exhibiting reduced pigmentation, though this trend was not statistically significant.

**Conclusion:**

Comprehensive characterization of the dermoscopic and histopathological features of BCC enhances lesion differentiation. AI-assisted lesion size and pigmentation analysis, combined with histopathological evaluation, improves diagnostic precision. Further studies with larger cohorts are needed to validate these findings and refine classification criteria.

## 1 Introduction

Basal cell carcinoma (BCC), arising from keratinocytes or their precursor cells, is the most prevalent form of skin cancer globally, with increasing incidence rates highlighted in large population studies (1). BCC typically manifests as flesh-colored to pink, pearly papules or nodules, often featuring ulceration, crusting, telangiectatic vessels, and rolled or raised borders. BCC presents several clinical subtypes, with nodular, superficial, and morpheaform being the most common (2). Although rarely metastatic, BCC can cause significant local destruction and disfigurement, underscoring its importance in dermatological research. Key risk factors for BCC include chronic ultraviolet (UV) radiation exposure, fair skin, genetic predispositions (e.g., xeroderma pigmentosum, basal cell nevus syndrome), male gender, immunosuppressive states, and environmental toxins such as arsenic (3).

Despite its slow progression, untreated BCC can escalate to more severe forms with low metastatic rates. Early diagnosis leads to an excellent prognosis, but delays can result in significant morbidity. Recent advances in therapy have markedly improved outcomes even for advanced cases (4). The diagnostic process includes detailed dermoscopic evaluation and confirmatory biopsy, classifying BCC into low-risk or high-risk categories, which guide treatment decisions. Recent advancements in imaging and genetic profiling have enhanced diagnostic accuracy, improving patient management (5).

Dermoscopic patterns vary by subtype: nodular BCC shows arborizing vessels and shiny white areas; superficial BCC features fine telangiectasia and blue-gray dots; morpheaform BCC has porcelain-white regions and ulceration; and infiltrative BCC exhibits prominent vessels and ulceration (6). Histopathology confirms the diagnosis, showing peripheral palisading of basaloid cells among other features, with variations in invasion depth observed across subtypes (7). Variations in invasion depth among BCC subtypes have been noted, with nodulocystic and aggressive forms presenting greater depths, while superficial BCCs show minimal invasion. Nodular BCCs tend to exhibit moderate depths, particularly in chronically sun-exposed areas like the neck (8).

Treatment strategies are adapted based on tumor stage, location, and subtype, ranging from surgical excision with defined margins to systemic therapies for advanced stages (9). Optimal surgical margins depend on the lesion's risk factors, with 3-mm margins typically sufficient for low-risk lesions, whereas high-risk or recurrent tumors may require 4–6 mm margins or Mohs micrographic surgery to minimize recurrence (10).

Advancements in artificial intelligence (AI) have enhanced the diagnostic precision of computer-aided dermoscopic and histopathological analysis for BCC. AI-assisted image processing enables quantitative assessment of lesion morphology, aiding in the differentiation of BCC subtypes and improving diagnostic consistency. In dermatopathology, AI-driven image analysis has the potential to reduce variability in histopathological interpretation and streamline workflow efficiency by providing objective, reproducible measurements (11–16).

Recent bibliometric analyses indicate a shift from traditional surgical methods like Mohs micrographic surgery toward less invasive techniques such as Hedgehog pathway inhibitors and photodynamic therapy, reflecting a move toward targeted therapies (17). Concurrently, the evolution of AI-based analyses now enables the precise differentiation of BCC, which is also the main focus of our study, from other skin cancers like melanoma, utilizing hyperspectral imaging to detect variations in biological molecules such as melanin, hemoglobin, and oxyhemoglobin, thus expanding the capabilities of traditional dermatoscopic data (18).

This study aims to investigate the dermoscopic and histopathological characteristics of BCC and assess the correlation between pigmentation patterns and tumor depth. By utilizing AI-assisted computerized image analysis, we seek to provide a quantitative and objective assessment of lesion morphology, enhancing the accuracy of BCC classification and contributing to more effective clinical decision-making.

## 2 Materials and methods

### 2.1 Study design and study population

This retrospective observational study included 41 patients with a total of 42 histopathologically confirmed BCC lesions. Patients underwent clinical examination and surgical excision at Gaziantep Nizip State Hospital between April 2023 and February 2025. Histopathological evaluations, including the preparation and analysis of biopsy specimens, were conducted at Gaziantep 25 Aralik State Hospital. The study primarily focused on patients with facial BCC lesions, but also included a small number of cases from non-facial regions such as the scalp, neck, and axilla. To ensure data quality and consistency, patients with poor-quality dermoscopic images, incomplete medical records, or a history of extensive prior treatments, such as surgery or cryotherapy, that could influence lesion analysis were excluded. Additionally, patients diagnosed with other concurrent skin malignancies were not included to maintain a specific focus on BCC. Furthermore, patients identified through routine dermatological screenings during the annual Euromelanoma campaign were also included. This initiative, aimed at raising skin cancer awareness and facilitating early diagnosis, contributed to detecting BCC cases in asymptomatic individuals and those with early-stage lesions.

### 2.2 Data collection, preparation, and evaluation

Data collection was conducted with meticulous attention to detail to ensure consistency and reliability. Within the structured clinical framework, demographic data, including age and gender, as well as clinical history, such as disease duration and prior occurrences of BCC, were systematically recorded. Lesion characteristics, including size and location, were also documented. High-resolution dermoscopic images were acquired to facilitate comprehensive analysis and correlation of BCC features with patient outcomes. Dermoscopic imaging was performed using a DermLite DL5 (DermLite, Carlsbad, CA, USA) device interfaced with an iPhone 15 Pro Max (Apple Inc., Cupertino, CA, USA) at 10× magnification, enabling detailed visualization of lesion morphology. To optimize image clarity and preserve vascular structures, images were captured with minimal pressure, utilizing ethanol as an immersion fluid to prevent air bubble interference. For lesions exceeding the field of view of a single dermoscopic frame, multiple high-resolution images were acquired and merged to reconstruct the entire lesion.

**Figure 1:**
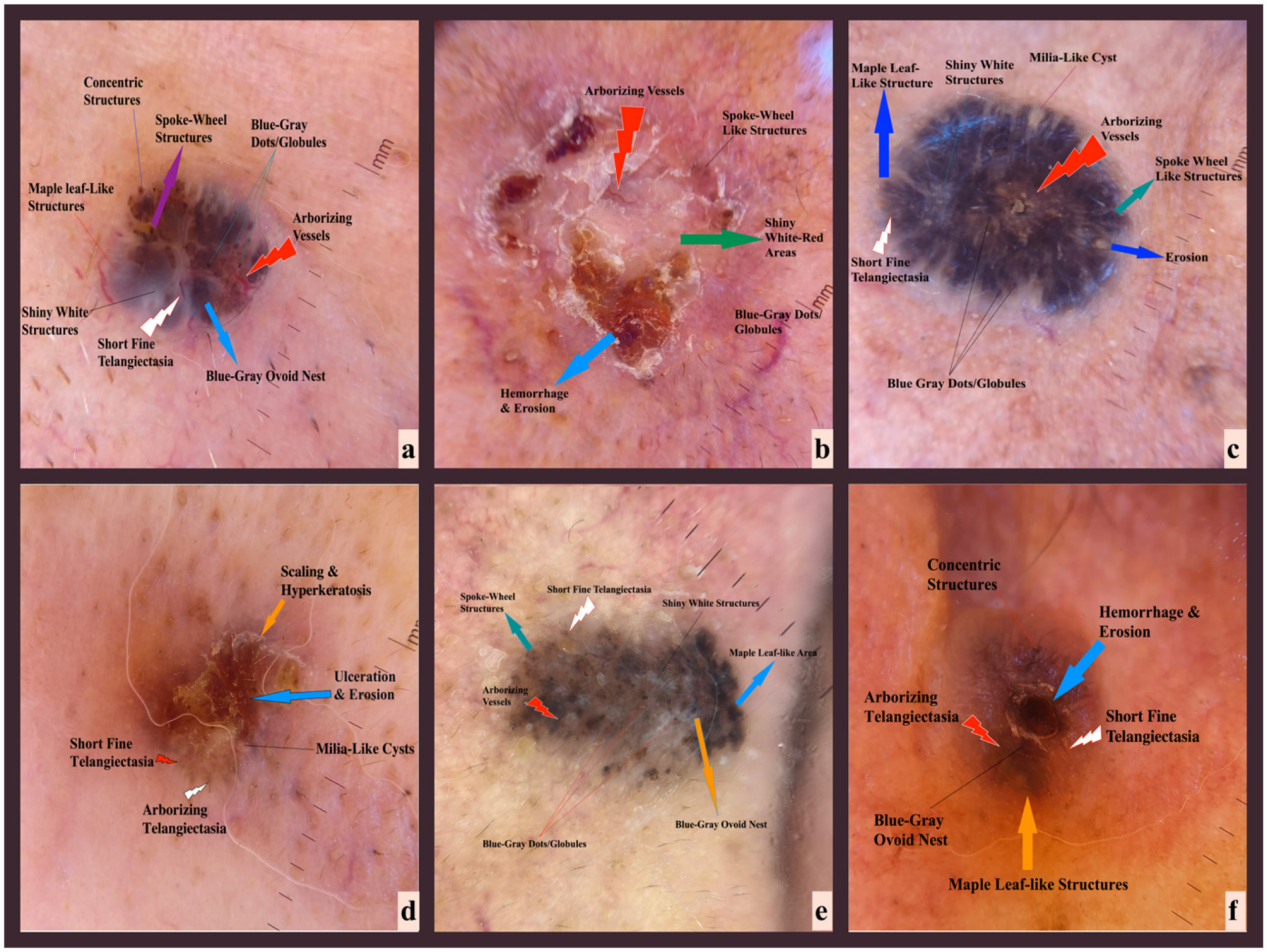
Representative dermoscopic features of different BCC subtypes from the study cohort. Dermoscopy images illustrating the characteristic features of different histological subtypes of BCC from patients included in this study. **(a)** Nodular BCC: Blue-gray ovoid nests and arborizing vessels. **(b)** Superficial BCC: Blue-gray dots, shiny white structures, and spoke-wheel structures. **(c)** Mixed (Nodular + Superficial) BCC: Combination of arborizing vessels and multiple pigmented structures. **(d)** Infiltrative BCC: Ulceration, hemorrhage, concentric structures, and arborizing telangiectasias. **(e)** Micronodular BCC: Blue-gray ovoid nests with fine arborizing vessels. **(f)** Adenoid BCC: Short fine telangiectasias and spoke-wheel structures.

Demographic and clinical data were retrieved from hospital records to analyze potential associations between patient characteristics and BCC subtypes. Macroscopic and dermoscopic images were standardized in terms of lighting and distance before being securely uploaded to a Google Drive (Google LLC, Mountain View, CA, USA) repository, providing authorized researchers with coordinated access for analysis. Dermoscopy evaluations focused on identifying pigmented structures, vascular patterns, and morphological features, ensuring precise and consistent assessments (19). The characteristic dermoscopic features of different BCC subtypes observed in this study are illustrated in Figure 1. Clinical examinations were performed by a dermatologist, while two independent dermatopathologists evaluated tumor characteristics, including histological subtype and depth. Additionally, an AI specialist applied image processing algorithms to refine lesion assessment and integrated these findings with clinical and histopathological data for enhanced diagnostic correlation.

**Figure 2:**
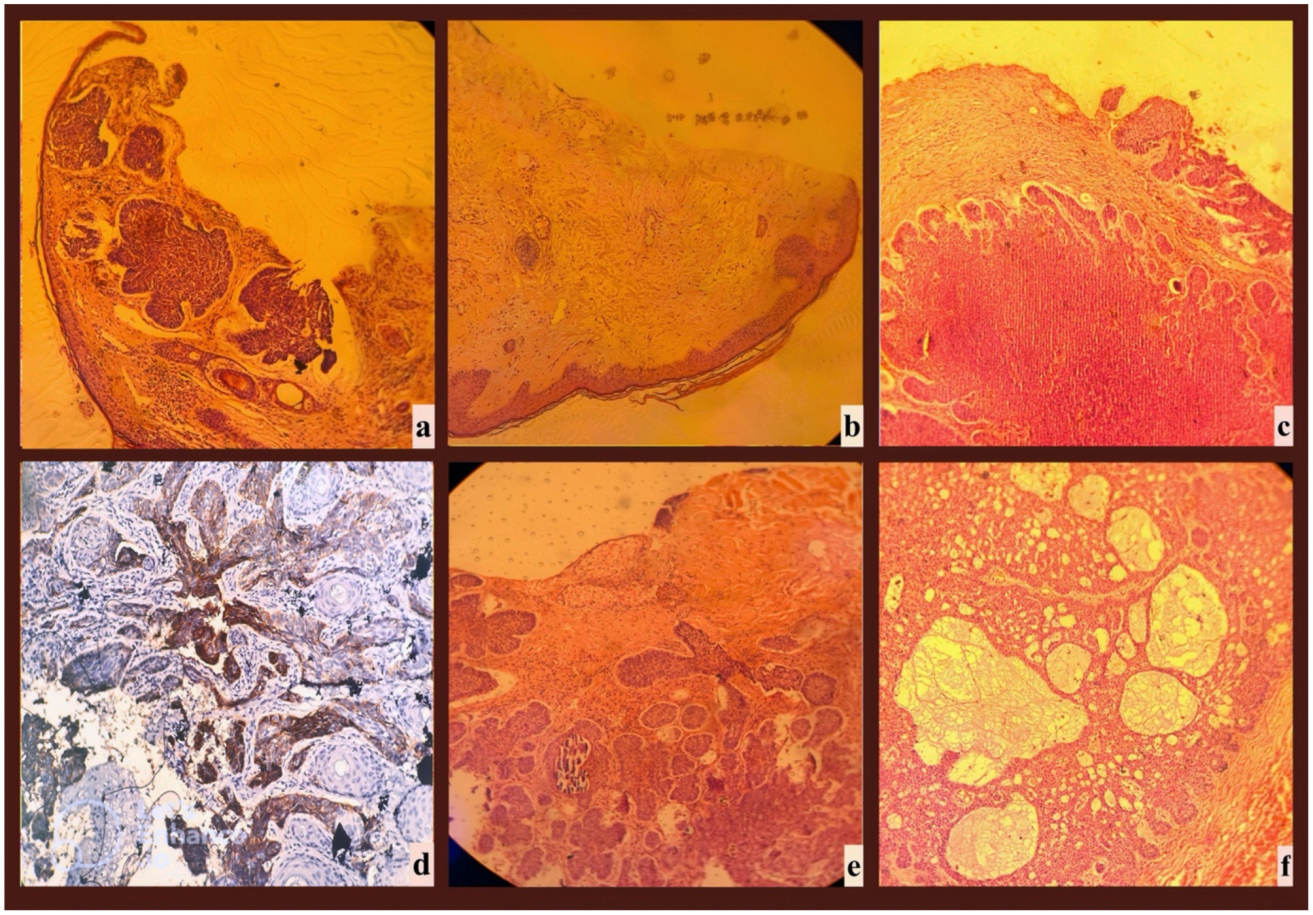
Histopathological Correlates of the BCC Cases Presented in Figure 1. Histopathological images corresponding to the same patients presented in Figure 1, demonstrating the distinct histological subtypes of BCC. Images **(a–c, e, f)** were stained with hematoxylin and eosin (H&E), while image **(d)** was stained using immunohistochemistry, most likely with Ber-EP4, to highlight epithelial tumor cells. All images were captured at 10× magnification. **(a)** Nodular BCC presents as large, basaloid tumor islands with peripheral palisading and stromal retraction artifacts. The presence of desmoplastic stroma and epidermal ulceration indicates progressive lesion growth, distinguishing it from superficial and infiltrative subtypes. **(b)** Superficial BCC is characterized by tumor islands confined to the epidermis with horizontal proliferation. The absence of deep invasion and mild stromal reaction differentiates it from other subtypes. **(c)** Mixed-type BCC exhibits both nodular and superficial components, with large basaloid nests infiltrating the dermis and smaller tumor buds within the epidermis. Peripheral palisading and stromal retraction artifacts support this diagnosis. **(d)** Infiltrative BCC is characterized by thin, irregular strands of basaloid tumor cells infiltrating the dermis, rather than forming well-defined nodular structures. In this section, Ber-EP4 immunohistochemical staining has been applied to enhance visualization of the epithelial tumor component. The presence of peritumoral clefting and a desmoplastic stromal reaction further supports the diagnosis of an aggressive histological subtype that may require wider surgical excision margins. **(e)** Micronodular BCC is composed of small, deeply invasive tumor islands with an infiltrative growth pattern. Dense fibrotic stroma and peripheral palisading are present, highlighting its aggressive nature and high recurrence risk. **(f)** Adenoid BCC is a rare variant showing cribriform and glandular-like structures within basaloid tumor nests. Reticulated spaces and stromal cystic degeneration distinguish it from other subtypes, necessitating differentiation from adenoid BCC.

The correlation between dermoscopic features and histopathological findings in BCC was systematically analyzed. Key dermoscopic patterns—such as blue-gray ovoid nests, arborizing telangiectasias, and shiny white structures—were matched with corresponding histopathological characteristics, including basaloid tumor islands, dilated stromal vessels, and dense fibrosis (Table 1). The histopathological images corresponding to the dermoscopic findings in Figure 1 are provided in Figure 2, demonstrating distinct BCC subtypes and their microscopic features.

**Table 1:** Dermoscopic features and their histopathological correlates in basal cell carcinoma.

**Dermoscopy feature**	**Histopathological correlation**
Blue-gray ovoid nests	Basaloid tumor islands with peripheral palisading.
Blue-gray dots and globules	Melanin-laden basaloid cells and melanophages within the tumor mass.
Maple leaf-like structures	Pigmented basaloid tumor lobules extending into the dermis.
Spoke-wheel structures	Radial arrangement of basaloid cells with high melanin content.
Concentric structures	Irregular tumor nests surrounded by fibrotic stroma.
Arborizing telangiectasias	Dilated, tortuous blood vessels within the fibrotic tumor stroma.
Short fine telangiectasia	Superficial vascular proliferation with minimal fibrosis.
Multiple erosions	Loss of epidermal integrity, with superficial ulceration.
Ulceration	Deep epidermal loss with necrotic tumor debris.
Shiny white-red areas	Stromal fibrosis and increased collagen deposition.
Shiny white structures	Dense fibrosis in the dermis, associated with aggressive tumor behavior.
Hemorrhage	Ruptured blood vessels and extravasated erythrocytes within the tumor stroma.
Scale	Hyperkeratosis and parakeratosis due to chronic irritation.
Milia-like cyst	Keratin-filled cystic spaces within the tumor mass.

Summary of dermoscopic features observed in basal cell carcinoma and their corresponding histopathological findings. The relationships presented are based on systematic reviews of dermoscopic and histopathological correlations in BCC. Data are adapted from Reiter et al. (6).

Surgical excision of BCC lesions was performed under local anesthesia using the elliptical excision technique, ensuring complete tumor removal with a standardized 2 mm clinical margin to minimize recurrence risk. The excision depth extended into the subcutaneous fat to achieve adequate clearance. Hemostasis was achieved using electrocautery to control intraoperative bleeding and optimize the surgical field. Following excision, layered suturing was employed to support wound healing and improve cosmetic outcomes. Deep dermal sutures were placed using absorbable material to reduce tension, while superficial sutures were applied for epidermal alignment and scar minimization. Excised specimens were immediately fixed in 10% buffered formalin and oriented for histopathological evaluation to confirm margin clearance and tumor subtype. Cases with positive margins or high-risk histopathological features were further assessed for re-excision or adjunctive therapy as needed.

Histopathological evaluation was performed following standard protocols. Biopsy specimens were initially fixed in 10% buffered formalin, embedded in paraffin wax, and sectioned at 4 μm thickness. Routine histological assessment was conducted using Hematoxylin and Eosin (H&E) staining. To ensure accurate differentiation of BCC from other cutaneous neoplasms, a panel of immunohistochemical markers was applied. BerEp4 and Bcl-2 were used to confirm the epithelial origin of the tumor and distinguish BCC from squamous cell carcinoma, as BCC exhibits strong BerEp4 and Bcl-2 positivity, whereas squamous cell carcinoma is typically negative for these markers. Epithelial membrane antigen and carcinoembryonic antigen were employed to differentiate BCC from adnexal tumors, given their variable expression patterns in sweat gland carcinomas and other adnexal neoplasms. CD10 expression was evaluated to further characterize BCC, as it has been associated with infiltrative and aggressive subtypes. S100 staining was performed to exclude melanocytic lesions, particularly malignant melanoma, which typically exhibits strong S100 positivity. Ki-67, a proliferation marker, was utilized to assess tumor growth activity and potential aggressiveness. All BCC lesions were classified into nodular, superficial, infiltrative-morpheaform, micronodular, or mixed subtypes based on their architectural growth patterns. In cases with mixed histological features, all relevant subtypes were documented to ensure precise classification.

**Figure 3:**
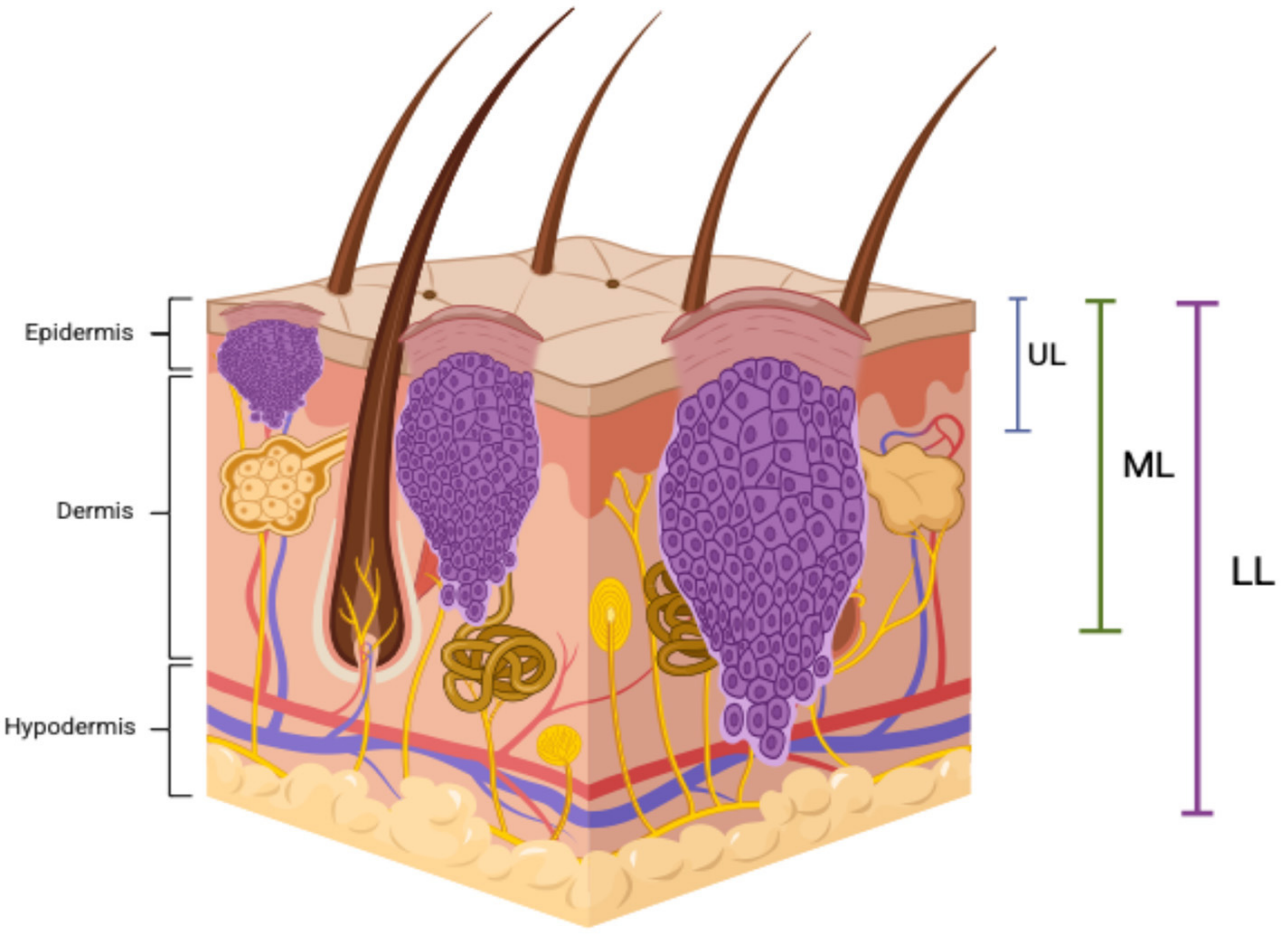
Schematic representation of tumor depth categories in basal cell carcinoma. A schematic illustration of tumor depth classification in BCC. The upper layer (UL, 0.1–0.2 cm) includes tumors infiltrating the epidermis and papillary dermis. The middle layer (ML, 0.3–0.4 cm) represents tumor extension into the upper two-thirds of the reticular dermis. The lower layer (LL, >0.5 cm) corresponds to infiltration into the lower third of the reticular dermis and hypodermis. This visualization reflects the progressive dermal invasion of BCC and its relevance to risk stratification. This figure was created using BioRender.com.

Microscopic evaluations were conducted using a CX21 Olympus microscope (Olympus Corporation, Tokyo, Japan) to assess histopathological features and classify BCC subtypes. Tumor architecture, cellular morphology, stromal characteristics, and peritumoral changes were systematically examined. Tumor depth measurements were performed using a micrometer-integrated microtome, enabling precise sectioning and stratification of tumor infiltration levels. Depth was categorized into three layers: upper (UL, 0.1–0.2 cm), middle (ML, 0.3–0.4 cm), and lower (LL, >0.5 cm), based on the extent of dermal invasion. This classification provided a standardized framework for evaluating tumor aggressiveness and potential therapeutic implications. A schematic representation of these tumor depth categories in BCC is illustrated in Figure 3.

### 2.3 Computerized image processing with AI-assisted quantitative analysis

To assess the pigmentation percentage and tumor area in BCC lesions, a computerized image analysis method was employed. Initially, a consulting dermatologist manually delineated the lesion boundaries and annotated pigmented structures within dermoscopic images using Procreate (Savage Interactive Pty. Ltd., Tasmania, Australia) on an iPad (9th generation, Apple Inc., Cupertino, California, USA). This manual annotation ensured accurate identification of clinically relevant pigmented regions, minimizing segmentation errors during subsequent image analysis.

After manual delineation, the annotated images were processed using Python and the OpenCV library to extract quantitative lesion parameters. Unlike fully automated segmentation methods, this approach combined expert-driven manual annotation with AI-assisted quantitative analysis to enhance measurement accuracy. The first step involved calculating the pixel-to-millimeter conversion ratio based on the millimeter scale embedded in the image. By referencing this scale, the millimeter value per pixel was determined. Lesion contours were extracted from the manually annotated regions using OpenCV's contour detection function [cv2.findContours()], ensuring that only the pre-defined tumor margins were analyzed. Subsequently, mask images were processed, contours were detected, and their areas were computed. The obtained pixel-based areas were then converted to square millimeters using the established pixel-to-millimeter conversion ratio.

**Figure 4:**
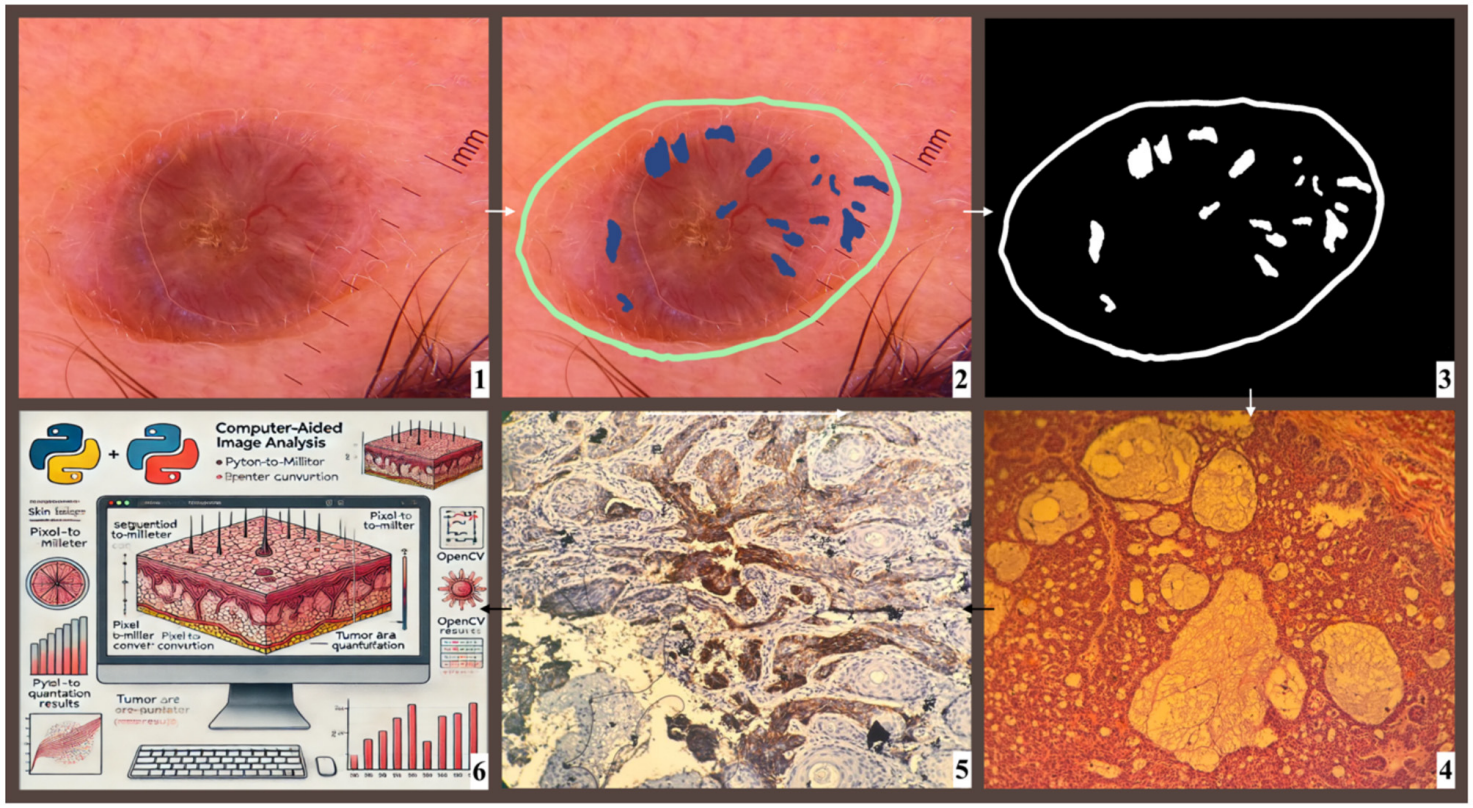
Workflow of computer-aided analysis and histopathological correlation in BCC diagnosis. This schematic illustration depicts the integrated diagnostic workflow utilized for evaluating BCC lesions in this study. (1) Dermoscopy Image Acquisition: A high-resolution polarized dermoscopic image of a suspected BCC lesion is obtained. (2) Manual Annotation: The lesion border and pigmented structures are manually annotated by a dermatologist using digital tools to ensure accurate segmentation. (3) Automated Image Processing: Thresholding and color-based segmentation techniques are applied to isolate annotated pigmented areas from non-pigmented background using OpenCV. (4) Histopathological Examination: The excised lesion undergoes conventional processing and hematoxylin and eosin (H&E) staining for assessment of tumor architecture and cellular morphology. (5) Immunohistochemical Confirmation: Tumor cells are stained with Ber-EP4, a marker that confirms the epithelial origin of BCC and differentiates it from other skin malignancies such as squamous cell carcinoma. (6) AI-Based Quantitative Analysis: Python and OpenCV algorithms are implemented to convert pixel data to metric measurements, detect lesion contours, and calculate tumor area and pigmentation percentage for objective evaluation.

This approach was a critical step in ensuring precise tumor size measurements and enhancing the accuracy of the analysis process. The Python and OpenCV-based method enabled automated and reproducible assessments, significantly improving the reliability and consistency of dermoscopic evaluations. The workflow of the entire computer-aided dermoscopic and histopathological analysis process is illustrated in Figure 4.

To standardize pigmentation measurements and ensure comparability across different lesions, the Pigmentation Coverage Ratio (PCR) was introduced as a quantitative parameter. The pigmentation percentage was calculated using the following formula:


$$\begin{array}{l} Pigmentation\;Coverage\;Ratio=\frac{Pigmented\;Area\;\left(mm2\right)}{Total\;Lesion\;Area\;\left(mm2\;\right)} \end{array}$$

where the total lesion area was measured within a standardized magnification field, excluding the horny layer of the epidermis.

To quantify pigmented areas within the lesion, color-based thresholding was applied to the manually annotated pigment regions using OpenCV's inRange() function. This step ensured that only dermatologist-defined pigmented areas were measured, avoiding artifacts and background noise.

To analyze the relationship between lesion size, pigmentation patterns, and histopathological findings, lesion areas were classified into three groups based on their dimensions. Lesions measuring ≤ 15 mm^2^ were considered small, those between 16–50 mm^2^ were classified as medium-sized, and those exceeding 50 mm^2^ were categorized as large. All dermoscopic images were standardized in terms of lighting, distance, and resolution before processing. The quantification of pigmentation was independently performed by two evaluators to ensure consistency and minimize measurement bias.

### 2.4. Ethical approval statement

The study received approval from the Bezmialem Vakif University Non-Interventional Ethics Committee (E-54022451-050.04-178629). It was conducted following the Declaration of Helsinki and Good Clinical Practice guidelines, ensuring rigorous adherence to ethical principles and privacy laws.

### 2.5 Statistical analysis

All statistical analyses were performed using IBM SPSS Statistics 28.0 (IBM Corp., Armonk, NY, USA), with a significance threshold of *p* < 0.05. Categorical variables were summarized as frequencies (n) and percentages (%), while numerical variables were expressed as mean ± standard deviation (SD). The Fisher-Freeman-Halton exact test was used to compare categorical variables due to the presence of multiple subcategories with small sample sizes. The relationships between numerical variables were analyzed using Pearson's correlation coefficient (*r*) for linear associations and Spearman's rank correlation coefficient (ρ) for nonparametric relationships. The correlation between dermoscopic pigmentation percentage and tumor depth was assessed using both Pearson's and Spearman's correlation analyses, while associations between tumor size categories, histopathological subtypes, and dermoscopic features were examined using the Fisher-Freeman-Halton exact test. All analyses were conducted at a 95% confidence level, and statistical significance was defined as *p* < 0.05.

## 3 Results

### 3.1 Demographic and clinical characteristics

Our study included 41 patients diagnosed with BCC, comprising 42 distinct lesions. The average patient age was 67.1 years, with a slight male predominance (56.1% male, 43.9% female), consistent with known demographic trends in BCC (Table 2). Most patients were over 60 years of age.

**Table 2:** Demographic and clinical characteristics of basal cell carcinoma patients.

**Variable**	**Description**
Total patients	*n =* 41
Total lesions	*n =* 42
**Age (years)**	
21–40 years	*n =* 0 (0%)
41–60 years	*n =* 17 (41.5%)
60–80 years	*n =* 16 (39.0%)
>80 years	*n =* 8 (19.5%)
**Gender**	
Male	*n =* 23 (56.1%)
Female	*n =* 18 (43.9%)
Duration of BCC (years)	3.49
**Location of lesions**	
Nose	*n =* 18 (42.9%)
Cheek	*n =* 6 (14.3%)
Upper lip	*n =* 5 (11.9%)
Medial canthus	*n =* 4 (9.5%)
Infraorbital	*n =* 4 (9.5%)
Others (ear, vertex, frontal, neck, axilla)	*n =* 6 (14.3%)
**Histopathological subtypes**	
Superficial	*n =* 8 (19.0%)
Nodular	*n =* 12 (28.6%)
Micronodular BCC	*n =* 1 (2.4%)
Adenoid basal cell carcinoma	*n =* 12 (28.6%)
Mixed (nodular + superficial)	*n =* 6 (14.3%)
Infiltrative type	*n =* 3 (7.1%)
Recurrence	*n =* 1
Tumor Area (mm^2^)	Average: 29.26 (±28.14)
**Tumor depth (mm)**	
Upper layer (UL, 0.1–0.2 cm)	*n =* 23 (54.76%)
Middle layer (ML, 0.3–0.4 cm)	*n =* 12 (28.57%)
Lower layer (LL, >0.5 cm)	*n =* 7 (16.67%)

This table summarizes the demographic and clinical characteristics of patients diagnosed with BCC, including age and gender distribution, lesion locations, histopathological subtypes, and tumor dimensions. The findings indicate a higher prevalence in males and older adults, with the nose being the most commonly affected site. Nodular BCC and adenoid BCC were the most frequently observed histopathological subtypes. Tumor depth analysis revealed that most lesions were confined to the upper and middle layers of the skin, highlighting the importance of early diagnosis to prevent deeper tissue invasion.

**Figure 5:**
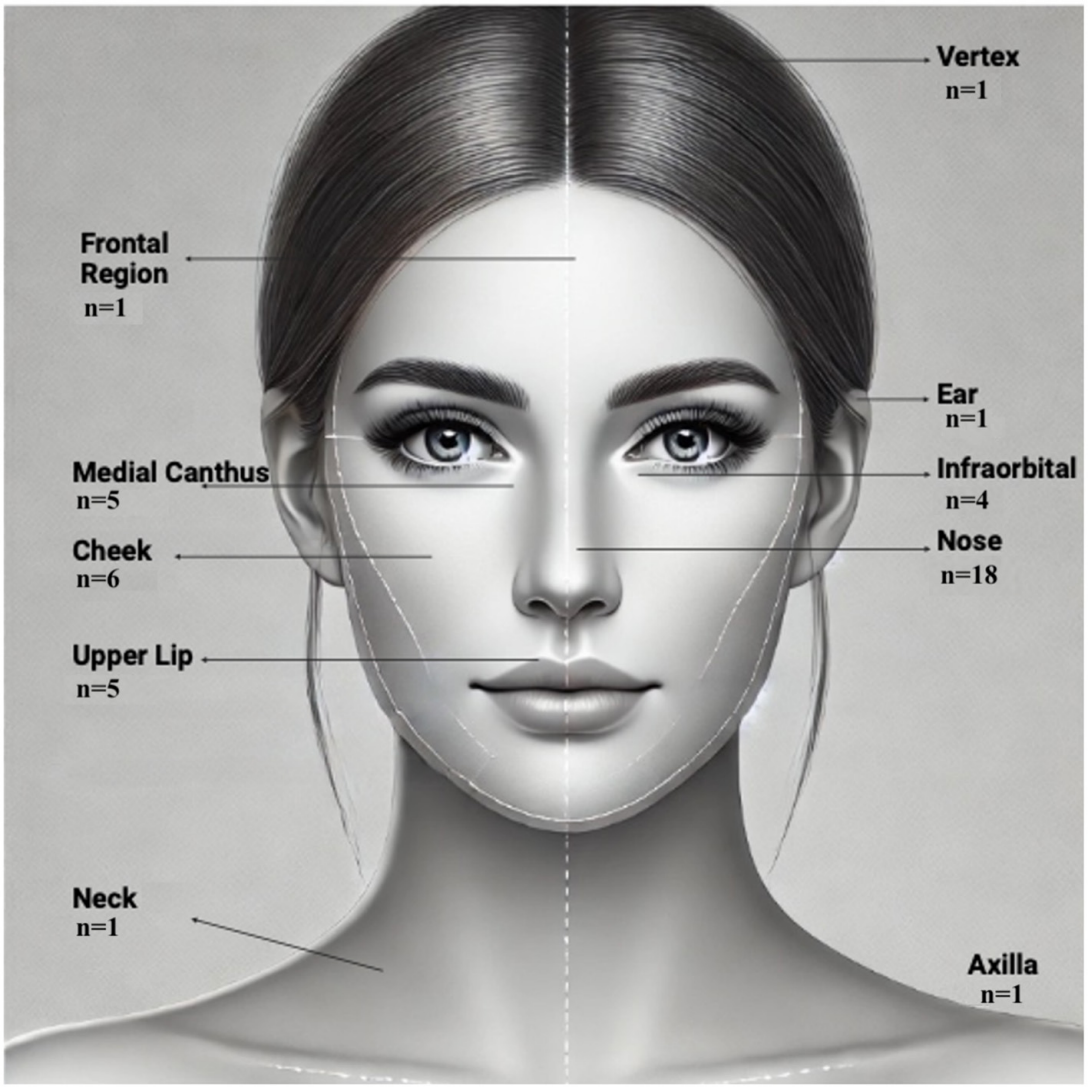
Anatomical distribution of BCC lesions in the study cohort. Schematic representation of lesion localization across the cohort (*n* = 42). The majority of lesions were located on the nose (*n* = 18, 42.9%), followed by the cheek (*n* = 6, 14.3%), upper lip (*n* = 5, 11.9%), and medial canthus (*n* = 5, 11.9%). Less frequent sites included the infraorbital area (*n* = 4, 9.5%), frontal region, ear, vertex, neck, and axilla (*n* = 1 each, 2.4%).

The most frequently affected anatomical region was the nose, followed by the cheek, upper lip, and medial canthus. Lesions were primarily located on the face, with a smaller proportion involving extrafacial regions such as the ear, vertex, neck, and axilla (Figure 5).

Histopathologically, nodular and adenoid subtypes were the most prevalent, while superficial, mixed, micronodular, and infiltrative forms were also observed. The average tumor area was 29.26 mm^2^ (±28.14). Most lesions were limited to the UL and ML of the skin, while a smaller portion extended into the LL, emphasizing the importance of early diagnosis to prevent deeper invasion (Table 2).

### 3.2 Correlation of dermoscopic features with histopathological subtypes and distribution across tumor size categories in basal cell carcinoma

**Figure 6:**
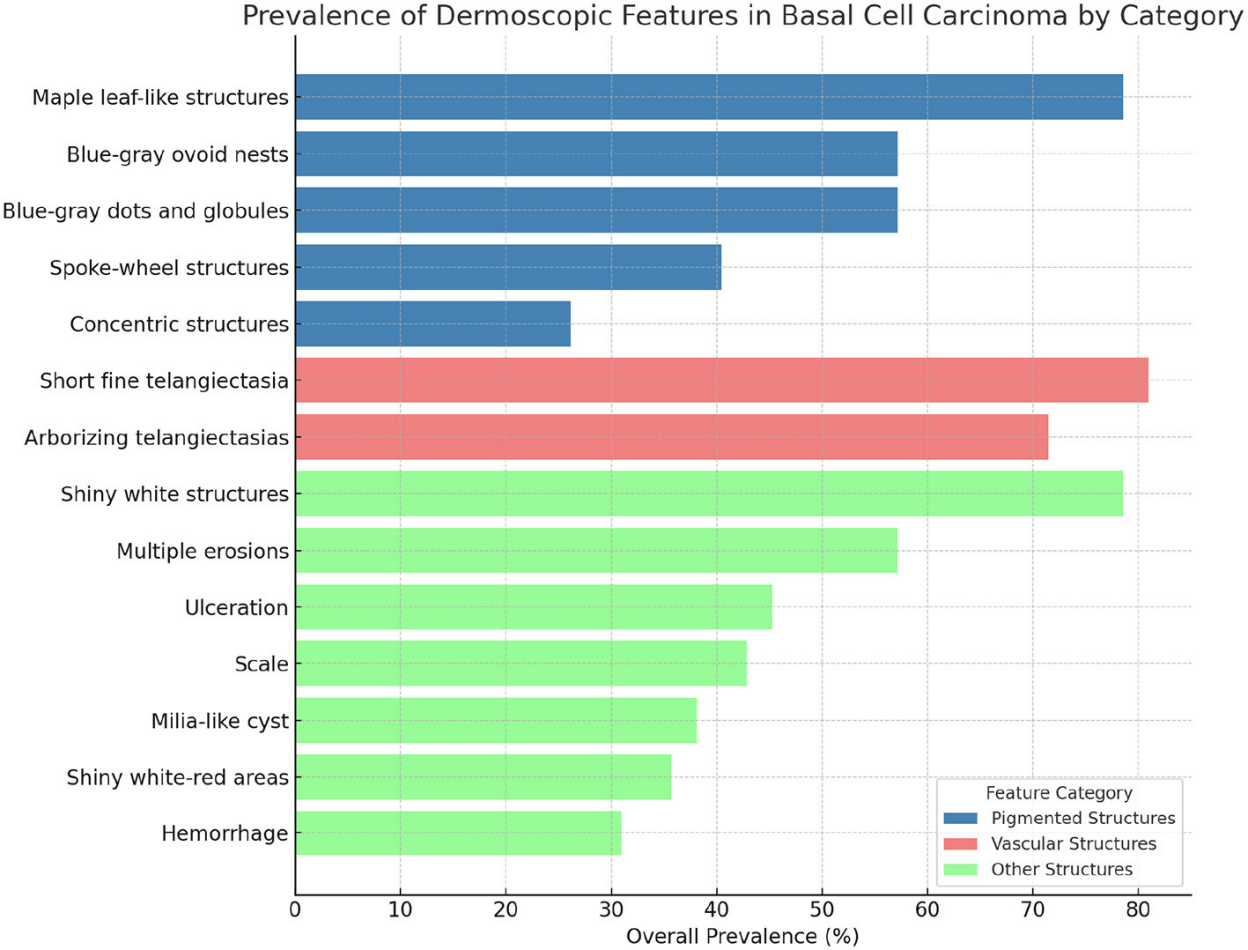
Prevalence of dermoscopic features in basal cell carcinoma. The figure illustrates the prevalence of dermoscopic features in basal cell carcinoma, categorized into Pigmented Structures, Vascular Structures, and Other Structures. Pigmented Structures are depicted in dodger blue, Vascular Structures in crimson, and Other Structures in dark khaki. These color distinctions enhance visual differentiation between categories, emphasizing the heterogeneity of dermoscopic features in basal cell carcinoma.

Dermoscopic features were analyzed in relation to histopathological subtypes and tumor size (*n* = 42). As shown in Figure 6 and Table 3, pigmented structures such as blue-gray ovoid nests and maple leaf-like areas were most frequently observed in mixed and micronodular BCC subtypes. Notably, maple leaf-like structures showed a statistically significant correlation with mixed BCC (*p* = 0.013).

**Table 3:** Correlation of dermoscopic features with histopathological subtypes in basal cell carcinoma.

**Category**	**Dermoscopic feature**	**Overall prevalence (%)**	**Strongest correlated subtype**	**Prevalence in subtype (%)**	***P*-value**
Pigmented Structures	Blue-gray ovoid nests	57.14%	Mixed Type	80.00%	0.113
	Blue-gray dots and globules	57.14%	Micronodular Type	100.00%	0.369
	Maple leaf-like structures	78.57%	Mixed Type	100.00%	**0.013**
	Spoke-wheel structures	40.48%	Micronodular Type	100.00%	0.479
	Concentric structures	26.19%	Infiltrative Type	50.00%	0.700
Vascular Structures	Arborizing telangiectasias	71.43%	Mixed Type	100.00%	0.403
	Short fine telangiectasia	80.95%	Micronodular Type	100.00%	0.465
Other Structures	Multiple erosions	57.14%	Infiltrative Type	100.00%	0.623
	Ulceration	45.24%	Infiltrative Type	100.00%	0.648
	Shiny white-red areas	35.71%	Infiltrative Type	50.00%	0.977
	Shiny white structures	78.57%	Micronodular Type	100.00%	0.843
	Hemorrhage	30.95%	Nodular Type	42.86%	0.722
	Scale	42.86%	Nodular Type	57.14%	0.777
	Milia-like cyst	38.10%	Micronodular Type	100.00%	0.503

This table summarizes the correlation between dermoscopic features and histopathological subtypes of BCC, categorized into pigmented, vascular, and other structural features. Maple leaf-like structures and arborizing telangiectasias were most frequently associated with mixed-type BCC, while blue-gray dots, globules, and spoke-wheel structures were exclusive to micronodular BCC. Ulceration and multiple erosions were prominent in infiltrative BCC. P-values indicate the statistical significance of these associations, highlighting the diagnostic value of dermoscopy in BCC classification.

Vascular features, particularly arborizing and short fine telangiectasias, were also common—both present in over 70% of cases—though their associations with histological subtypes were not statistically significant. Structural indicators of aggressive behavior, including ulceration and multiple erosions, were predominantly observed in infiltrative BCC, while shiny white structures were more prevalent in micronodular subtypes, potentially indicating stromal fibrosis.

**Figure 7:**
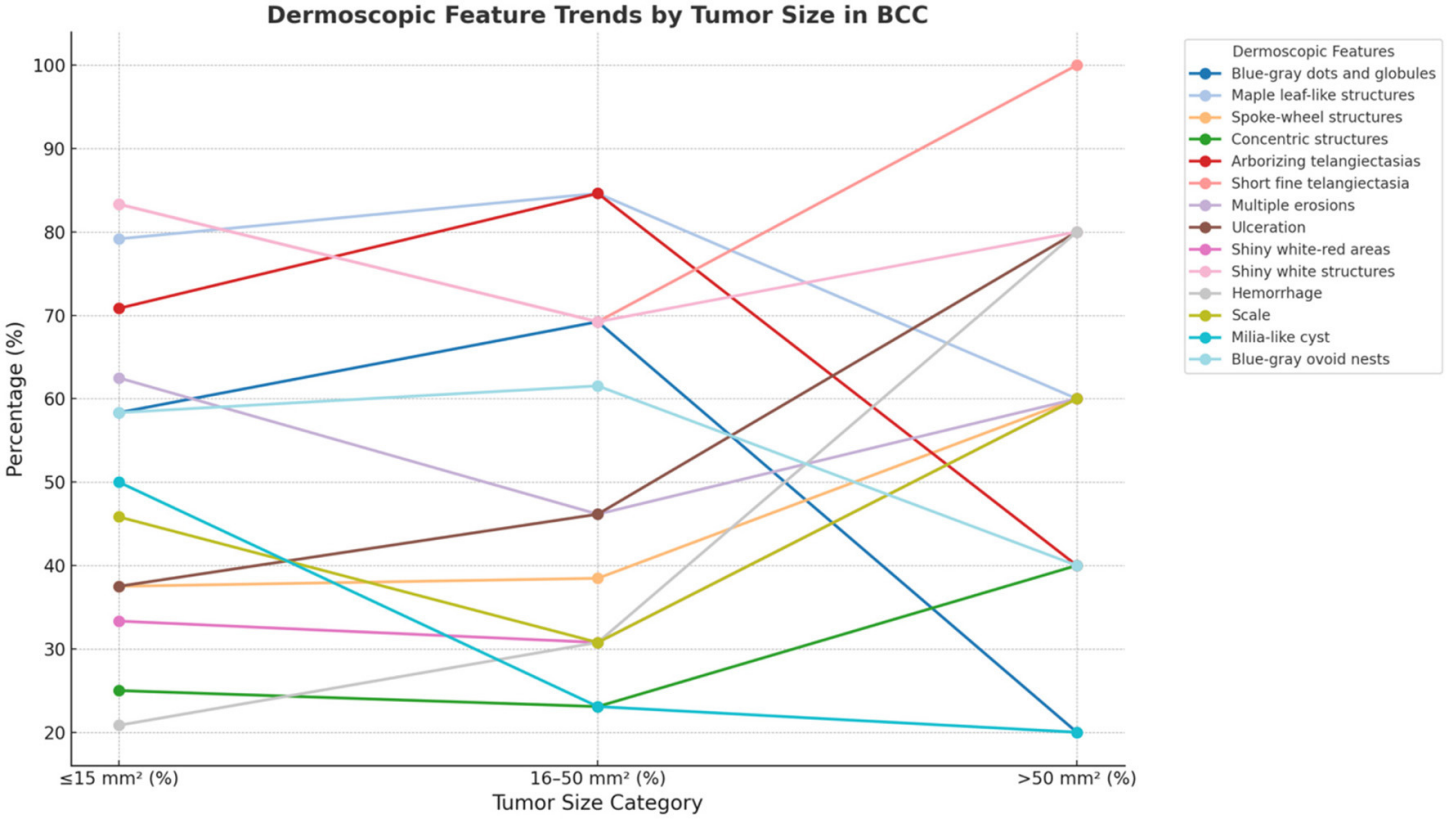
Distribution of dermoscopic features by tumor size in basal cell carcinoma. This graph illustrates the variation of dermoscopic features across different tumor size categories in BCC. Each line represents a distinct dermoscopic feature, demonstrating its percentage prevalence within tumors sized ≤ 15, 16–50, and >50 mm^2^. The trends highlight how certain features may become more or less prevalent as the tumor size increases, providing insights into the morphological changes associated with the progression of BCC. This visual analysis aids in understanding the diagnostic characteristics that correlate with tumor growth.

When stratified by tumor size ( ≤ 15, 16–50, >50 mm^2^), the distribution of dermoscopic features revealed a clear trend (see Table 4 and Figure 7). Pigmented structures such as blue-gray ovoid nests and globules were more common in smaller tumors and declined as tumor size increased. In contrast, ulceration and hemorrhage were significantly more prevalent in larger tumors (*p* = 0.034 for hemorrhage), suggesting an association with lesion progression and vascular changes. Certain features, including short fine telangiectasia and shiny white structures, were consistently present across all size groups.

**Table 4:** Distribution of dermoscopic features across tumor size categories in basal cell carcinoma.

**Dermoscopic feature**	** ≤ 15 mm^2^ (%)**	**16–50 mm^2^ (%)**	**>50 mm^2^ (%)**	***P*-value**
Blue-gray dots and globules	58.33%	69.23%	20.00%	0.165
Blue-gray ovoid nests	58.33%	61.54%	40.00%	0.699
Maple leaf-like structures	79.17%	84.62%	60.00%	0.519
Spoke-wheel structures	37.50%	38.46%	60.00%	0.637
Concentric structures	25.00%	23.08%	40.00%	0.750
Arborizing telangiectasias	70.83%	84.62%	40.00%	0.171
Short fine telangiectasia	83.33%	69.23%	100.00%	0.298
Multiple erosions	62.50%	46.15%	60.00%	0.625
Ulceration	37.50%	46.15%	80.00%	0.221
Shiny white-red areas	33.33%	30.77%	60.00%	0.477
Shiny white structures	83.33%	69.23%	80.00%	0.606
Hemorrhage	20.83%	30.77%	80.00%	**0.034**
Scale	45.83%	30.77%	60.00%	0.481
Milia-like cyst	50.00%	23.08%	20.00%	0.185

This table presents the distribution of various dermoscopic features according to tumor size in BCC. Tumors are categorized into three groups based on their size: ≤ 15, 16–50, and >50 mm^2^. The prevalence of each dermoscopic feature is displayed for these size categories, along with corresponding p-values indicating statistical significance. Some features, such as short fine telangiectasia and shiny white structures, were observed consistently across all tumor sizes, whereas others, like hemorrhage and ulceration, were more frequently associated with larger tumors (>50 mm^2^). These findings provide insights into the potential correlation between tumor size and dermoscopic characteristics, which may aid in the clinical assessment of BCC progression. Bold values indicate dermoscopic features with statistically significant variation across tumor size categories (*p* < 0.05).

These findings support the role of dermoscopy in evaluating tumor subtype and progression. The transition from pigmented to vascular and ulcerative features with increasing tumor size reinforces the utility of dermoscopic monitoring in BCC management.

### 3.3 Comparison of average dermoscopic pigmentation percentages by tumor depth

We assessed the relationship between tumor depth and dermoscopic pigmentation percentage across three histological layers: UL, ML, and LL. As summarized in Table 5, pigmentation levels showed a decreasing trend with increasing depth, with the highest average pigmentation in the UL (17.86%) and the lowest in the LL (11.69%).

**Table 5:** Comparison of average dermoscopic pigmentation percentages by tumor depth in basal cell carcinoma.

**Tumor depth category**	**Average pigmentation (%) ± SD**	**Pearson correlation (*r*)**	**Pearson *p*-value**	**Spearman correlation (ρ)**	**Spearman *p*-value**
Upper Layer (UL) (0.1–0.2 cm)	17.86% ± 14.78	−0.158	0.473	−0.151	0.491
Middle Layer (ML) (0.3–0.4 cm)	15.42% ± 18.27	−0.345	0.272	−0.333	0.290
Lower Layer (LL) (0.5–0.6 cm)	11.69% ± 11.15	−0.348	0.445	−0.612	0.144

The analysis explores the correlation between tumor depth and dermoscopic pigmentation percentages in BCC, divided into categories based on depth: Upper Layer (0.1–0.2 cm), Middle Layer (0.3–0.4 cm), and Lower Layer (0.5–0.6 cm). The results indicate a trend of decreasing pigmentation as tumor depth increases, with the most notable decrease observed in the Lower Layer. While the correlations are not statistically significant, they suggest that deeper tumor layers tend to have less pigmentation, a finding that could be relevant for diagnostic approaches. Additional research with larger datasets is recommended to verify these trends and assess their clinical implications.

**Figure 8:**
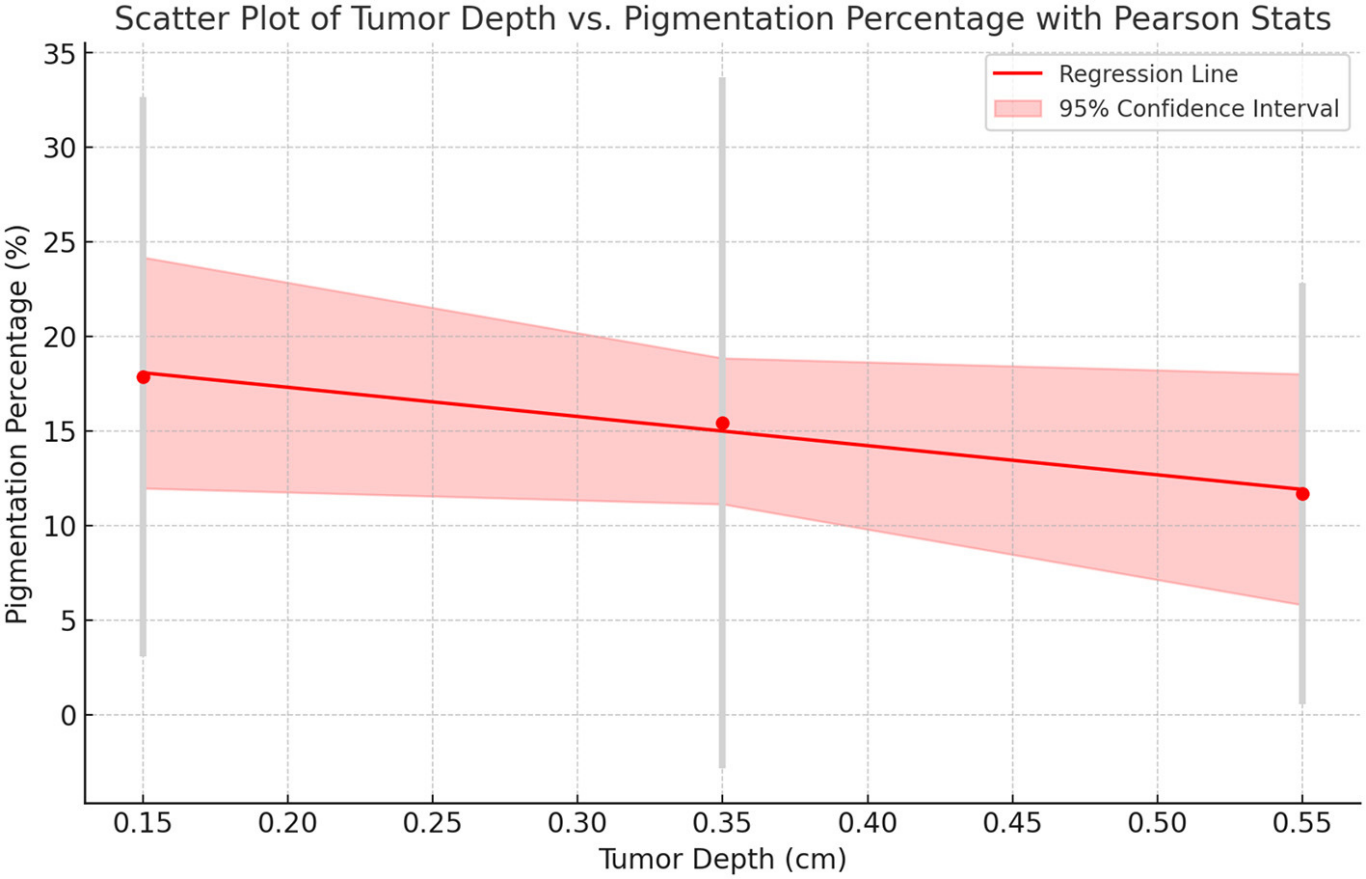
Scatter plot with spearman correlation: tumor depth vs. pigmentation. This scatter plot illustrates the relationship between tumor depth (cm) and pigmentation percentage (%), incorporating Spearman's correlation coefficient (ρ) to assess the strength and direction of the association. The red dashed line represents the trend, while individual data points indicate observed pigmentation levels across different tumor depth categories. The Spearman correlation values and corresponding *p*-values are annotated for each depth category. A negative correlation trend suggests a decrease in pigmentation percentage with increasing tumor depth.

While the correlation was not statistically significant, both Pearson and Spearman analyses indicated a consistent inverse relationship between depth and pigmentation, particularly in the lower layer (ρ = −0.612, *p* = 0.144). This trend is visually depicted in Figure 8, supporting the hypothesis that deeper tumors may exhibit reduced pigmentation.

Although further studies with larger cohorts are needed, these findings may have diagnostic relevance in distinguishing superficial BCCs from more invasive subtypes.

## 4 Discussion

This retrospective study analyzed excisional biopsy specimens from 41 BCC patients with clinical and dermoscopic evaluations at 42 distinct lesion sites between April 2023 and February 2025. Our findings align with the global increase in BCC incidence, primarily due to cumulative UV exposure. Contributing factors include ozone depletion, prolonged outdoor activities, and insufficient protective clothing, which are particularly relevant in our region due to intense solar radiation and widespread outdoor occupations such as agriculture and livestock farming (20).

The mean age of patients was 67.1 years, with a gender distribution of 56.1% males and 43.9% females, indicating a slight male predominance. This aligns with regional studies highlighting age and sex as significant BCC risk factors (21, 22). However, our relatively balanced gender ratio, compared to earlier reports of stronger male predominance, may reflect changing BCC epidemiology. While increased occupational UV exposure in middle-aged men contributes to higher incidence, older age groups show less gender disparity due to cumulative sun damage affecting both sexes similarly. Additionally, increased healthcare awareness and improved access may lead to earlier diagnosis in females. These observations underscore the evolving epidemiology of BCC and highlight the importance of preventive strategies, particularly in populations at high risk of chronic UV exposure (23, 24).

Our study identified an average diagnostic delay of 3.5 years, particularly longer for scalp and extrafacial lesions. This finding aligns with a Spanish study reporting an average delay of 19.79 months, associated with older age and no prior BCC diagnosis (25). Similarly, studies from the US emphasize that diagnostic delays often stem from patients underestimating symptoms and healthcare accessibility challenges, further exacerbated during the COVID-19 pandemic (26).

The high prevalence of BCC lesions in UV-exposed areas, particularly the nose and other facial regions, underscores the critical role of chronic sun exposure in BCC pathogenesis. This finding aligns with prior studies indicating a twofold increase in BCC risk in chronically sun-exposed sites, irrespective of histological subtype or Fitzpatrick skin type (27).

Dermoscopy categorizes BCC structures as pigmented, vascular, and non-pigmented/non-vascular. Blue-gray ovoid nests and blue-gray dots/globules were present in 57.14%, while maple leaf-like structures appeared in 78.57% of lesions—both significantly higher rates than previously reported by Lallas et al. who found these features less frequently (28). Arborizing telangiectasias (71.43%) and short fine telangiectasias (80.95%) were also more prevalent in our cohort compared to Emiroglu et al. (42.9% and 15.3%, respectively). This discrepancy might result from differences in dermoscopic techniques, lesion localization, or the proportions of histopathological subtypes studied. Vascular patterns in BCC distinctly differ from normal skin, exhibiting complex branching rather than simple linear telangiectasias typical of benign lesions (29). Ulceration (45.24%) and multiple erosions (57.14%) were prominent, particularly in infiltrative BCC, exceeding previously reported rates. The high prevalence of shiny white structures (78.57%) in micronodular BCC suggests stromal fibrosis, supporting the significance of vascular and structural dermoscopic assessment in predicting BCC subtype and guiding clinical management (30).

In this study, the diagnosis of BCC was predominantly confirmed through excisional biopsy combined with local surgical excision. This approach allowed accurate determination of histological subtype and tumor depth, minimizing reliance on small skin biopsies. Alam et al. demonstrated that excisional biopsies frequently identify additional histological subtypes missed by smaller biopsies, highlighting the value of complete excision for precise histopathological evaluation (31). Although active surveillance may be suitable for older patients with low-risk BCC due to its low mortality (32), our preference was surgical management, particularly due to the high prevalence of nodular and adenoid subtypes, which are classified as low-risk variants but still require complete excision for accurate histopathological evaluation and recurrence prevention. Future studies should evaluate the necessity of surgery for low-risk lesions in elderly populations. Histopathological subtypes identified included nodular, adenoid, superficial, mixed (nodular + superficial), infiltrative, and micronodular forms, with nodular and adenoid subtypes being most common. Compared to previous reports, our study indicated a higher frequency of adenoid BCC but fewer infiltrative and micronodular cases (33).

Our analysis revealed correlations between dermoscopic features and histopathological subtypes. Popadić and Brasanac reported short fine telangiectasias as diagnostic for superficial BCC, whereas in our cohort, these were predominantly associated with micronodular BCC. Similarly, arborizing telangiectasias showed the strongest association with mixed-type BCC, suggesting vascular features may not be subtype-specific (34). Lallas et al. highlighted blue-gray ovoid nests and dots/globules as typical of pigmented nodular BCC (28); however, in our study, ovoid nests correlated most with mixed-type BCC, and dots/globules exclusively with micronodular BCC. Maple leaf-like and spoke-wheel structures, classically associated with superficial BCC, were frequent in mixed and micronodular subtypes, indicating these features are not exclusive markers. Additionally, ulceration and erosions predominated in infiltrative BCC, supporting their role as indicators of aggressiveness. Song et al. emphasized variability of dermoscopic features based on patient age and lesion site, stressing that no single dermoscopic characteristic definitively indicates a specific histopathological subtype (35). Future studies should investigate how age and anatomical location influence these dermoscopic variations. These findings highlight the necessity of integrating dermoscopic, clinical, and histopathological assessments for accurate diagnosis and treatment planning.

We classified tumor areas into three categories—small ( ≤ 15 mm^2^), medium (16–50 mm^2^), and large (>50 mm^2^)—using computerized image processing, allowing detailed analysis of dermoscopic variation by tumor size. Ulceration and hemorrhage occurred significantly more often in larger tumors, supporting prior reports associating these features with increased vascularization and invasiveness (36–38). Conversely, pigmented structures such as blue-gray dots/globules were more common in smaller tumors, suggesting pigmentation as an early diagnostic indicator. Arias-Rodriguez et al. similarly reported increased pigmentation in early-stage BCC (39). Although most BCCs are amelanotic (2), dermoscopy can detect subtle pigmentation in up to 30% of cases (28, 40). This finding aligns with histopathological data showing increased melanocyte density in pigmented BCC (41). Our cohort had an average pigmentation percentage of 14.79%, with pigmentation decreasing as tumor size increased. Recognizing pigmentation patterns is crucial for differentiating BCC from melanoma, which typically exhibits atypical networks and blue-whitish veils (42). Reflectance confocal microscopy studies confirm that melanoma pigmentation involves atypical melanocytes, whereas BCC pigmentation results from melanophages (43). While tumor size is a more significant prognostic factor for melanoma, its importance in BCC emphasizes early diagnosis and precise measurement for accurate staging, prognosis, and treatment decisions (44).

In our study, dermoscopic pigmentation decreased with increasing tumor depth, averaging 17.86% in the UL, 15.42% in the ML, and 11.69% in the LL. Although a negative correlation (Spearman's ρ = −0.612, *p* = 0.144) was observed, it was not statistically significant. This suggests greater pigmentation in superficial tumors; however, larger studies are needed to confirm clinical relevance. Park et al. similarly reported higher pigmentation in less aggressive BCC subtypes (45). Surkov et al. used diffuse reflectance spectroscopy to demonstrate melanin variability with tumor depth (46), and Negrutiu et al. emphasized linking dermoscopic features to tumor invasion depth for improved diagnostic accuracy (47). Pyne et al. (8) and Wetzel et al. (48) highlighted the importance of invasion depth in tailored surgical management, noting aggressive variants exhibit deeper invasion. Furthermore, Unar et al. found deeper tumors had higher recurrence rates, underscoring the necessity of precise depth assessment for effective treatment (49). Our findings reinforce the clinical importance of assessing pigmentation and depth to refine risk stratification and surgical decision-making.

A retrospective study conducted over 24 months demonstrated significant cost savings (74.0%−75.3%) with outpatient BCC surgeries under local anesthesia compared to traditional operating room procedures (50). Our findings align with this, emphasizing that integrating cost-effective surgical methods with advanced dermoscopic and histopathological assessments can optimize diagnostic accuracy and resource utilization. Enhancing diagnostic and therapeutic capabilities at secondary care centers can improve patient outcomes by reducing delays caused by geographical and systemic healthcare barriers.

## 5 Conclusion

This study provides a comprehensive evaluation of dermoscopic and histopathological features of BCC, emphasizing correlations between pigmentation patterns and tumor depth. Our findings highlight a high BCC incidence in a low-latitude region (36°28′E, 36°38′N), characterized by intense UV exposure and Fitzpatrick skin types II–IV. The absence of universal dermoscopic markers exclusive to specific subtypes underlines the morphological heterogeneity of BCC, reinforcing the necessity of integrating dermoscopic and histopathological evaluations. Additionally, increased tumor size correlated with prominent invasive features such as ulceration, hemorrhage, and stromal changes (shiny white-red areas), whereas pigmentation, particularly blue-gray dots/globules and ovoid nests, decreased. Pigmentation also diminished progressively with greater tumor depth, suggesting its potential utility in distinguishing superficial from invasive lesions. Further studies involving larger patient cohorts are necessary to validate this diagnostic pattern.

Considering the increasing global prevalence of BCC, early and accurate diagnosis is crucial for reducing morbidity and improving outcomes. Advanced computer-aided image analysis, including Python and OpenCV-based algorithms, shows significant promise for lesion assessment and classification. Future research should refine automated quantification techniques, particularly regarding pigmentation analysis, within broader and more diverse populations. Expanding AI-assisted dermoscopy in clinical practice could enhance diagnostic accuracy and facilitate personalized management of BCC.

## 6 Limitations

This study has several limitations. Its retrospective design may introduce selection bias, as inclusion depended on available excisional biopsy specimens and dermoscopic images rather than random patient selection. The relatively small sample size also limits the generalizability of the findings. Using excisional biopsies may lead to classification bias, particularly in mixed histological patterns where certain subtypes could be underrepresented. Additionally, tumor depth measurements may be influenced by interobserver variability, potentially affecting consistency. Although computerized image analysis quantified pigmentation and tumor depth objectively, accuracy depends on algorithm quality, image resolution, lighting conditions, and dermoscopy equipment calibration, introducing possible measurement bias. To overcome these limitations, future studies should employ prospective, multicenter designs with standardized imaging protocols and larger patient cohorts. Advances in AI-driven segmentation and automated feature extraction could further reduce observer bias and improve lesion assessment accuracy, supporting enhanced risk stratification and treatment planning for BCC.

## Data Availability

The original contributions presented in the study are included in the article/supplementary material, further inquiries can be directed to the corresponding author.
